# Sociodemographic determinants of vaccination and willingness to pay for COVID-19 vaccines in Hungary, results of a cross-sectional online questionnaire

**DOI:** 10.1186/s12889-024-18797-z

**Published:** 2024-05-16

**Authors:** Zsuzsanna Beretzky, Valentin Brodszky

**Affiliations:** Department of Health Policy, Corvinus University of Budapest, Fővám Tér 8, 1093 Budapest, Hungary

**Keywords:** COVID-19, Vaccine, Willingness to pay, Socio-demographic factors

## Abstract

**Background:**

Several different coronavirus disease (COVID-19) vaccines were authorized and distributed all over the world, including Hungary, but vaccination rates and acceptance of the different vaccines varied through 2021 and subsequent years. In Hungary Western vaccines and the Chinese and Russian vaccines were available in early 2021. Understanding preference and willingness to pay (WTP) for the COVID-19 vaccine could provide information for policy decision making to control the COVID-19 pandemic. We aimed to assess the socio-demographic factors influencing the COVID-19 vaccination and to analyse individual preferences for the available COVID-19 vaccines in Hungary.

**Methods:**

A cross-sectional online questionnaire survey was conducted between 25–05-2021 and 08–06-2021 exploring the vaccine acceptance and WTP for vaccination in the Hungarian general population. To assess the preferences towards the different vaccines available in Hungary at the time of the study, we used a multi-step WTP task.

**Results:**

Altogether 2,000 respondents filled out our survey, with the average age of 49.1 (SD = 15.3), out of whom 370 respondents (18.5%) stated that they already had a COVID-19 infection. Age above 65 years, male gender, higher level of education, higher income and residence in the capital or county seats were associated with a higher probability of vaccination. The average WTP ranged from 14.2 to 30.3 EUR for the different vaccine types.

**Conclusions:**

Males, respondents with higher education and income stated a higher WTP value for all vaccines. Better socioeconomic status increased both vaccination coverage and willingness to pay for vaccines.

## Introduction

Different vaccines were available worldwide at the time of our survey. Several different coronavirus (COVID-19) vaccines were authorized and distributed all over the world, but both vaccines developed and produced in the EU and one developed and produced in China and Russia were available in early 2021. Many factors, such as sociodemographic, psychological, sociological, environmental characteristic and indicators of anxiety, COVID-19 related fear may influence the willingness to vaccinate.

Vaccine rollout.

At the beginning of the pandemic, isolation and social distancing were regarded as the main measure against the infection, and since the availability of vaccines, vaccination has become the primary tool in the fight against the virus. To receive any vaccination an online or offline registration (per mail) was required from all citizens in Hungary towards the Office of the Prime Minister. Due to geopolitical situation of Hungary, vaccines have been available in this country very soon (spring 2021), compared to other continents and countries. The Hungarian government decided very early to purchase vaccines – at that time with no EU authorisation – from partners both from Russia and China. This might have been the reason for Hungary having been the second-best vaccinated nation in Europe in March–April 2021 – but these rates have not grown as fast as expected and the country fell back to place 20 in October 2021 with an average vaccination rate of 61,8% [47]. At the time of the survey five different vaccines: AstraZeneca (Vaxzevria), Moderna (Spikevax), Beijing CNGB BBIBP (CorV), Pfizer-BionTech (Comirnaty) and Gamaleya (Gam-Covid-Vac) refered to as AstraZeneca, Moderna, Sinopharm, Pfizer-Biontech, and Sputnik V. were available in Hungary, but choices on the type of the vaccine were limited at the beginning and differed depending on when and under which circumstances (administered by a general practitioner or received at central vaccination points) someone received the vaccine.

**Fig. 1:**
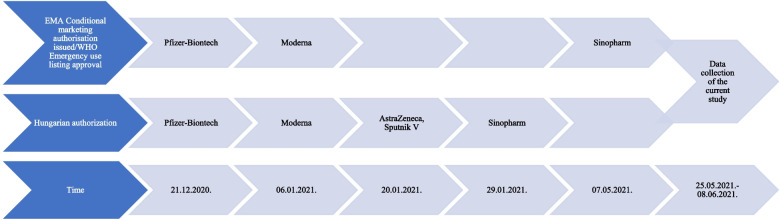
Vaccine authorization timeline

In most cases, if someone did not accept the specific vaccine they were offered, they had to wait, and it was not certain that the next vaccine offered would be their preferred vaccine. The perception of the effectiveness and the potential side effects of vaccines differed in the Hungarian population. At the time of the survey, negative news about possible risks and side effects related to the AstraZeneca vaccine were dominating the public opinion [1]. The Sputnik V. and Sinopharm were believed to be less effective, and their lack of European Medicines Agency (EMA) authorization also increased mistrust [2]. (Fig. 1).

As of January 2022, rega﻿rding the uptake of additional doses of vaccines, Hungary has already reached a comparably favourable position within the EU with a 64.1% of the population taking at least one dose of vaccine, which is higher than countries in the region such as Croatia (56.8%), Slovakia (50.9%), Slovenia (57.3%) and Romania (41.6%) [3]. COVID-19 vaccination hesitancy can be explained by multiple factors. One set of factors are the sociodemographic characteristics, for example ethnicity, gender, age, educational level, socio-economic status and religion.[4–7]. Another set of determinants can be the COVID-19 and lockdown-related fear and anxiety. A recent German study has shown that participants with higher fear of COVID-19 were more likely to be vaccinated [8], while a study from the Netherlands found increased fear of COVID-19 predicts vaccination willingness 14 months later, even when controlling for other factors [9]. In Japan, a longitudinal online study revealed that mental health conditions such as depression and generalized anxiety, and low level of fear of COVID-19 can be associated with unwillingness or indecision regarding COVID-19 vaccination [10].

### Willingness to pay (WTP) for vaccines

WTP estimates reveal individuals' values for goods or services, that have not been valued by the market such as vaccines, which also have large external effects and are most often provided or subsidized by public funding, hence they provide information for decision-makers on understanding population preferences. WTP provides valuable information on public preferences and the social value of an intervention through the summation of individuals’ WTP. It has been widely used to gain information on relative valuations for different treatment options for medical conditions, health care priority setting and among other healthcare interventions, vaccines [11]. WTP methods have been used in numerous studies worldwide to estimate the value of vaccinations, including hypothetical and actual COVID-19 vaccines [12–22]. WTP valuations are often influenced by respondents’ characteristics and methodological choices. In the context of COVID-19 vaccination, differences based demographic and socioeconomic determinants, such as age, gender, income and education were observed in previous studies [11, 16, 19, 23].

Our objective was to assess the sociodemographic factors that might influence vaccination and to identify the determinants of vaccination. Measuring population’s willingness to pay for the COVID-19 vaccine could help understand preferences towards vaccines and provides valuable information to aid policy interventions on vaccination and on the control the COVID-19 pandemic. Hence, this study also aimed to estimate the Hungarian populations’ preference for vaccines using the WTP method and to assess any potential differences between the attitudes towards the different types of vaccines available in Hungary at the time of the survey.

## Methods

### Data collection

The data for this study came from a large cross-sectional, internet based national survey, exploring the vaccine acceptance, willingness to pay for vaccination for COVID-19 on the Hungarian general population. The survey was conducted between 25–05-2021 and 08–06-2021 as an online questionnaire survey (with an approximately 30 minute completion time) among the Hungarian adult (age > 18) general population. At this time, lockdown rules were relaxed after the COVID-19 pandemic neared the end of its’ third wave (March-June 2021) in Hungary. The recruitment of the respondents was carried out by a survey company from members of an online panel. The online panel had over 150 thousand members who had voluntarily registered to complete surveys in return for earning survey points that could be later redeemed to various rewards (e.g. gift card, prizes). The target sample size was 2,000 and non-probabilistic quota sampling was used, aiming for representativeness in terms of sex, age, level of education, type of settlement and region.

All procedures performed in studies involving human participants were in accordance with the 1964 Helsinki declaration and its later amendments or comparable ethical standards. Permission for conducting the study was granted by the Research Ethics Committee of Corvinus University Budapest (reference No. KRH/109/2021). Respondents were informed that the participation in the survey was completely voluntary, their data would remain anonymous and would not be linked to personal information and used solely for scientific purposes. Respondents needed to provide their informed consent before the start of the survey.

### Questionnaire

The survey included questions on demographic and socio-demographic (such as age, gender, education, marital status, employment status, household size, monthly net household income, place of residence) factors, and respondents’ experience with previous COVID-19 infections. The questionnaire also included a question on whether the respondent has been vaccinated, or registered to get vaccinated, but did not receive the vaccine yet or was not vaccinated, nor registered. We also included a question regarding what type of vaccine the respondent got, if they stated that they were vaccinated. Respondents were asked about their willingness to pay for the vaccines that were available in Hungary at the time of the survey. The questionnaire also included scales and questionnaires to assess the quality of life, anxiety, and the COVID-19 related fear of the survey population, as we aimed to understand the possible connections between these factors and vaccination. The questions we used in our analysis were mandatory questions, hence we did not have missing data in the variables.

### Quality of life, anxiety, and fear

#### General Anxiety Disorder-7

The General Anxiety Disorder-7 (GAD-7) is commonly used for the assessment of generalized anxiety disorder; a questionnaire that examines the symptoms of the past two weeks. Answers are given a 4-point Likert scale (0–3), with a total score between 0 and 21. Final scores above 10 points are associated with moderate, above 15 points with severe anxiety disorder [24].

### Fear of COVID-19

The Fear of COVID-19 (FCV-19) Scale is seven-item scale, which can be used to assess fear of COVID-19 among the general population [25]. The questionnaire was validated in more than 15 different languages and populations [26–32]. We used the Hungarian validated version of the questionnaire [33]. Participants are asked to indicate their level of agreement with the statements using a five-item Likert-type scale. The answers were the following: “strongly disagree,” “disagree,” “neither agree nor disagree,” “agree,” and “strongly agree”. The minimum score possible for each question is 1, and the maximum is 5. A total score is calculated by adding up each item score (ranging from 7 to 35). Higher scores are associated with greater fear [25].

### Lockdown captivity

During the COVID-19 lockdown, travel and leisure activities were limited, the consequent losses in activities and changes in lifestyle has been challenging for many. The lockdown captivity scale was developed as a part of a wider research project on the travel-related burden experienced during the Covid-19 lockdown, and aims to conceptualize the lockdown captivity phenomenon [34]. The scale includes the following three statements: “Wish you could break out of the lockdown situation”, „Feel trapped by the lockdown situation”, and „Wish you could just run away” [34]. The response categories were ranging from “strongly disagree” to “totally agree” on a 7-point Likert scale.

### Fear of missing out

Fear of missing out (FOMO) has become an increasingly relevant social phenomenon, which became even more important when the COVID-19 pandemic and related lockdown periods have started. The FOMO scale measures anxiety that individuals experience when they miss out on rewarding experiences with others (e.g., going out with friends). The scale contained four statements which respondents were asked to rate on a 7-point Likert scale with answers ranging from “strongly disagree” to “totally agree.” and has been used and adapted in multiple countries [35, 36].

#### WTP

WTP is a decision theory that uses the methods of conditional evaluation and conditional response [37]. Using a multi-step WTP task, participants were asked about their willingness to pay for vaccines (AstraZeneca, Moderna, Sinopharm, Pfizer-Biontech, and Sputnik V.) available in Hungary at the time of the study. We only provided the name and the country of origin of the vaccine and did not display any further information (effectiveness, probability of potential side-effects), as we were interested in analysing how respondents think about vaccinations, based on their own information. Respondents got randomly assigned one of the available vaccines and asked to state how much they could and would certainly pay for the vaccine, after which they were asked to state the amount that they certainly would not be able to or willing to pay. By asking the respondents to identify all the amounts they would certainly pay and those that they would certainly not pay, we gained information on the range of values over which people are uncertain of [37]. The next step contained an open-ended question. The boundaries in the open-ended question were determined by the amounts the respondents had indicated in the previous two questions. In the following question all the other vaccines were presented to the respondents who were asked to indicate the amount they would be willing to pay for each in an open-ended question without any boundaries. The participants gave their willingness to pay values in Hungarian forint, which was then converted into euros using the average exchange rate of the Central Bank of Hungary in May 2021 (1 EUR = 354.2 HUF) [38]. To assess to which level participants were able to assert their preferences, we ranked each respondents’ WTP answers (in case of equal values expressed for two or more vaccines, we gave the same rank to the vaccines with the same WTP value) for the five vaccines and compared it to which vaccine they received for their first dose. At the time of our survey the choice on vaccine was partly limited in Hungary.

### Statistical analysis

The statistical analyses used the IBM SPSS Statistics 25.0 software. Statistical characteristics of nominal and quantitative variables were shown as descriptive statistics (arithmetic means and standard deviations), respectively. We conducted sub-group analysis in the 1) vaccinated, 2) non-vaccinated but registered to be vaccinated and 3) non-vaccinated and non-registered subgroups. We used non-parametric tests (Kruskal–Wallis test) to analyse the significance of the differences between groups as our scale variables had a non-normal distribution. To compare the nominal variables across the groups, we used crosstab analysis and reported the empirical significance of the chi square tests.

To explore the determinants of vaccination, we built a multinomial logistic regression model. The dependent variable was a variable expressing the vaccination status of the respondent. The variable expressed whether the participant was (1) vaccinated, (2) registered, but not vaccinated, or (3) non-vaccinated. The classification of respondents who were registered, but not vaccinated is ambiguous as there may be respondents among them who do not want to be vaccinated anymore, hence, to handle this uncertainty, we treated them as a separate group regarding their vaccination status. In our model, we selected the reference category as not vaccinated nor registered. To explore the determinants of vaccination, we included factors (gender, age over 65 years, living in the same household with someone over the age of 65, income over the median, settlement type, employment status, previous mandatory quarantine status and a control variable on previous COVID-19 infection, categorized GAD-7 and Fear of COVID-19 scores), and covariates (Fear of Missing Out and Lockdown captivity scores) in our model.

## Results

### Demographic characteristics of respondents

Overall, *N* = 2,421 started the questionnaire, the final sample contains *N* = 2,000 complete responses of the Hungarian adult general population (response rate: 82.6%). The average age of the respondents was 49.1 years (SD = 15.3). Almost half of the respondents were employed full time (*N* = 920 (46.0%)), while a quarter of the respondents were retired (*N* = 510 (25.5%)). Altogether 370 respondents (18.5%) stated that they already had a COVID-19 infection, which was higher the national rate with altogether 8.2% (798,147) inhabitants infected until the 15th of May 2021. The mean score on the FCV-19 was 13.9 (SD = 5.5), indicating that the respondents were affected by the COVID-19 infection and experienced fear (Table 1).

**Table 1:** Comparison of vaccinated and non-vaccinated sub-groups

Variables	Total sample	Vaccinated	Registered, but not vaccinated	Non-vaccinated	p*
	**N (%) or Mean (SD)**	**N (%) or Mean (SD)**			
Total	2,000 (100.0%)	1,374 (67.4%)	106 (5.3%)	547 (27.4%)	N/A
Age					
Age < 65	1,647 (82.4%)	1,045 (63.7%)	95 (5.8%)	507 (30.9%)	< 0.001
Age ≥ 65	353 (17.7%)	302 (85.6%)	11 (3.1%)	40 (11.3%)	
Age < 65 living in the same household with					
Age ≥ 65	684 (34.2%)	536 (78.4%)	20 (2.9%)	128 (18.7%)	< 0.001
Age < 65	1,316 (65.8%)	811 (61.6%)	86 (6.5%)	419 (31.8%)	
Gender					
Male	756 (37.8%)	554 (73.3%)	28 (3.7%)	174 (23.0%)	< 0.001
Female	1,244 (62.2%)	793 (63.7%)	78 (6.3%)	373 (30.0%)	
Education					
Primary	435 (21.8%)	253 (58.2%)	22 (5.1%)	160 (36.8%)	< 0.001
Secondary	900 (45.0%)	567 (63.0%)	52 (5.8%)	281 (31.2%)	
Tertiary	665 (33.3%)	527 (79.2%)	32 (4.8%)	106 (15.9%)	
Residence					
Capital	428 (21.4%)	320 (74.8%)	21 (4.9%)	87 (20.3%)	< 0.001
City	1,088 (54.4%)	729 (67.0%)	54 (5.0%)	305 (28.0%)	
Village	484 (24.2%)	298 (61.6%)	31 (6.4%)	155 (32.0%)	
Region					
Central Hungary	637 (31.9%)	468 (73.5%)	33 (5.2%)	136 (21.4%)	< 0.001
Northern Hungary	236 (11.8%)	163 (69.1%)	14 (5.9%)	59 (25.0%)	
Northern Great Plain	288 (14.4%)	168 (58.3%)	21 (7.3%)	99 (34.4%)	
Southern Great Plain	273 (13.7%)	167 (61.2%)	14 (5.1%)	92 (33.7%)	
Central Transdanubia	199 (10.0%)	126 (63.3%)	8 (4.0%)	65 (32.7%)	
Western Transdanubia	200 (10.0%)	134 (67.0%)	11 (5.5%)	55 (27.5%)	
Southern Transdanubia	167 (8.4%)	121 (72.5%)	5 (3.0%)	41 (24.6%)	
Income (missing: *n* = 355)					
< 300 000HUF (847 EUR)	869 (52.8%)	539 (62.0%)	49 (5.6%)	281 (32.3%)	< 0.001
≥ 300 000HUF (847 EUR)	776 (47.2%)	573 (73.8%)	37 (4.8%)	166 (21.4%)	
GAD-7 (0–21)^a^	4.5 (4.4)	4.2 (4.7)	6.5 (5.8)	5.0 (5.3)	< 0.001
FCV-19 (7–35)^a^	13.9 (5.5)	14.2 (5.3)	14.8 (6.6)	12.8 (5.8)	< 0.001
Fear of Missing Out (7–28)^a^	12.4 (7.5)	12.1 (7.3)	13.6 (8.0)	12.8 (8.1)	0.135
Lockdown Captivity (7–21)^a^	11.1 (5.5)	10.6 (5.3)	12.5 (5.3)	12.2 (6.0)	< 0.001

^*^Pearson's chi-squared test p values/Kruskal–Wallis test *p* values/

^a^Higher scores indicating greater level of fear and anxiety

### Comparison of vaccinated and non-vaccinated sub-groups

Altogether 1,374 (67.4%) respondents received at least the first dose of vaccine, while 106 (5.3%) have registered to be vaccinated, and 547 respondents (27.4%) did not get the vaccine and did not register to be vaccinated. Altogether 25.1% (*n* = 338) of the vaccinated respondents did not receive the second dose at the time of the study.

When comparing the vaccinated and non-vaccinated sub-groups, we found that the rate of vaccination was higher in the group aged above 65 years (85.6% vs. 63.7%, *p* < 0.001), and male respondents had a higher rate of vaccination than female respondents (73.3% vs.63.7%, *p* < 0.001), suggesting that they might be more willing to accept the vaccine (*p* < 00.1 in all cases). Education had a significant positive impact on vaccine acceptance: while in respondents with primary education only 58.2% was vaccinated, in those who have tertiary education the rate is 79.2% (*p* < 00.1). The vaccination rate was the highest in the capital city (74.8%) (*p* < 0.001). Higher income was associated with higher vaccination rate within the group with a monthly income over 300,000HUF (847 EUR) the rate of vaccinated respondents was 73.8%, compared to 62.0% in the group with a lower income level (*p* < 00.1). (Table 1) Vaccinated (14.2) and registered, but not vaccinated (14.8) respondents had a higher average FCV-19 scores than non-vaccinated respondents (12.8), indication that COVID-19 related fear, and vaccination may be associated with each other (*p* < 0.001). The physical symptoms of COVID-19 related fear (FCV-19 items 3; 6 and 7) were less frequently reported to cause problems than the anxiety-related items (FCV-19 item 5). Vaccinated and non-vaccinated were similar in terms of which FCV-19 items were most effected.

Lockdown Captivity scores were higher in those respondents who did not receive a vaccine yet, (*p* < 0.001) and the first item (“Wish you could break out of the lockdown situation”) was most affected with 29.8% of the respondents agreeing fully with the statement. GAD-7 scores were the lowest in the vaccinated subgroup (4.2 (SD = 4.7) indicating the lowest level of anxiety was observable among them (*p* < 0.001). Altogether 58.3% of the respondents reported at least some problem in item 1 (“Feeling nervous, anxious or on edge”) and 7.3% of the respondents reported daily problems in item 4 (“Trouble relaxing”). Mean FOMO scores were not significantly different across the three subgroups (*p* = 0.135).

### Regression analysis

The multinomial logistic regression showed that male gender (OR = 1.316), age over 65 years (1.647), living in the same household with someone over the age of 65 (1.370), higher than median income (1.913), residence in Budapest (1.622), employment statuses (working, student, retired, disability pensioner 1.776; 2.305; 3.380 and 2.853 respectively) and moderate or severe COVID-19 related fear (2.004) were significantly associated with higher probability of vaccination. Previous COVID-19 infection and stronger sense of being trapped in lockdown showed a significant, negative association with the probability of vaccination (0.536 and 0.922).

In the subgroup of registered, but not vaccinated respondents, less variables, age over 65 years, student status, previous experience of a mandatory quarantine, moderate or severe anxiety (GAD-7) and moderate or severe COVID-19 related fear had significant positive effect on the probability of vaccination (3.261, 6.664, 2.404, 1.624 and 1.769, respectively); the model fit was acceptable (*p* < 0.001). (Table 2).

**Table 2:** Regression models exploring the determinants of vaccination^a^

	Registered, but not vaccinated				Vaccinated			
	Sig	Exp(B)	95% Confidence Interval for Exp(B)		Sig	Exp(B)	95% Confidence Interval for Exp(B)	
			Lower Bound	Upper Bound			Lower Bound	Upper Bound
Intercept	< 0.001				0.357			
Female gender	0.420	1.255	0.723	2.177	**0.036**	0.760	0.588	0.982
Over 65 years of age	**0.055**	3.261	0.975	10.911	**0.072**	1.647	0.956	2.836
Living in the same household with someone over the age of 65	0.330	0.676	0.307	1.488	**0.065**	1.370	0.980	1.916
Income over the median income (300 000HUF/847 EUR)	0.453	1.212	0.733	2.005	**0.000**	1.913	1.488	2.460
*Residence (reference category: living in a town)*								
Living in Budapest	0.938	0.972	0.476	1.985	**0.010**	1.622	1.124	2.341
Living in a city, excluding Budapest	0.264	0.736	0.430	1.260	0.131	1.240	0.938	1.638
*Employment (reference category: unemployed)*								
Working	0.147	2.514	0.724	8.726	**0.028**	1.776	1.065	2.964
Student	**0.020**	6.664	1.351	32.880	**0.079**	2.305	0.907	5.856
Retired	0.363	2.093	0.426	10.269	**0.000**	3.380	1.773	6.442
Disability pensioner	0.247	2.791	0.491	15.868	**0.008**	2.853	1.308	6.223
Other employment status	0.664	1.370	0.331	5.666	0.702	0.888	0.483	1.632
Previously had COVID-19 infection	0.455	1.270	0.679	2.376	**0.001**	0.536	0.374	0.768
Previously had been in a mandatory quarantine	**0.007**	2.404	1.267	4.562	0.140	1.335	0.910	1.957
GAD7 categorized^b^	**0.066**	1.624	0.968	2.725	0.234	0.853	0.657	1.108
FCV-19^c^	**0.039**	1.769	1.031	3.038	**0.000**	2.004	1.491	2.692
Lockdown Captivity	0.415	0.978	0.926	1.032	**0.000**	0.922	0.897	0.947
Fear of Missing Out	0.601	1.011	0.971	1.052	**0.007**	1.028	1.008	1.049
Cox and Snell Pseudo R square: 0.154, Model Fitting Criteria Log Likelihood *p* value < 0.001								

^a^The reference category was "not vaccinated and did not register to be vaccinated",

^b^GAD scores were categorized into a binary variable, where scores below 10 (mild and moderate) were given a 0 value, all scores above a 1 value,

^c^FCV-19 scores were categorized into a binary variable, where scores below 13.5 were given a 0 value, all scores above a 1 value

### Willingness to pay

**Fig. 2:**
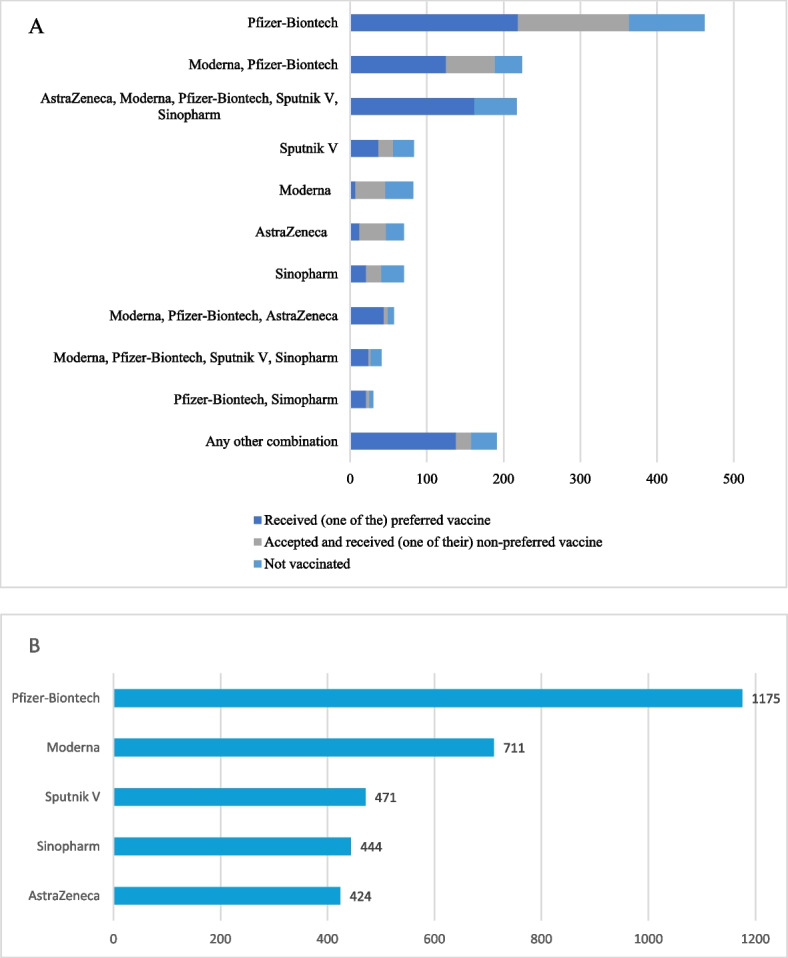
Preferences of vaccines based on WTP answers (n) **A** First preference of vaccine or vaccine-combinations (if several vaccines were chosen as first preference at the same time)based on WTP values **B** First preferred vaccine based on WTP results, where in case of equal WTP values, multiple vaccines are considered as first preference **A** Only includes respondents, who gave a WTP value higher than 0. Where multiple vaccines are listed. the same WTP values were expressed. The lines are mutually exclusive. **B** Only includes respondents, who gave a WTP value higher than 0. If the highest WTP value was expressed for more than one vaccine, all the vaccines with that WTP are considered first preferences. The lines are not mutually exclusive

Ranking the vaccines by WTP, most respondent preferred the Pfizer-Biontech vaccine in first place (*n* = 462, 23.1%). The second largest subgroup ranked Pfizer and Moderna vaccines in first place with same WTP value (*n* = 224, 11.2%), while the third (*n* = 217, 10.9%) ranked all vaccines with the same WTP value (higher, than 0). (Fig. 2).

Although the choice on which vaccine someone could receive was limited, people were able to assert their preferences. Altogether 71% (*n* = 975) of the vaccinated respondents expressed the highest WTP value to the vaccine they received. (Table 3) Out of those who received their first preferences 52% (*n* = 506) got the Pfizer-Biontech, 15% (*n* = 148) the Sinopharm, 13% (*n* = 130) the Sputnik V, 12% (*n* = 117) the AstraZeneca and 8% (*n* = 74) the Moderna vaccine. Altogether 170 respondents (12.4%) received their second preference, while 116 (8.6%) and 57 (4.2%) got their third and fourth choice as their first dose. Only 20 respondents (1.5%) got their least preferred vaccine based on their WTP answers; out of whom 9 respondents received the Sinopharm, 5 the AstraZeneca, 4 the Sputnik V and 2 respondents the Pfizer-Biontech vaccine.

**Table 3:** Comparison of WTP for vaccines between vaccinated, registered, and non-vaccinated subgroups (2021 EUR)

Vaccine^a^	WTP for vaccines, mean (SD) (EUR)				*p*	Ranked as highest WTP among vaccinated n (%)^b^	Vaccine recieved		
	**Total sample *n* = 2,000**	**Vaccinated *n* = 1,374**	**Registered, but not vaccinated *n* = 106**	**Non-vaccinated *n* = 547**			**Total sample *n* (%)**	**As first ranked vaccine n (%)**	**As second ranked vaccine n (%)**
**Pfizer-Biontech**	30.3 (52.3)	39.3 (58.3)	21.0 (29.2)	9.7 (28.8)	< 0.001	1,126 (83.6%)	549 (40.8%)	506 (92.2%)	30 (5.5%)
**Moderna**	24.4 (48.3)	31.6 (53.9)	16.2 (24.5)	8.1 (29.5)	< 0.001	734 (54.5%)	92 (6.8%)	74 (80.4%)	15 (1.6%)
**Sputnik V**	16.1 (37.9)	21.2 (44.2)	9.5 (14.7)	4.8 (13.8)	< 0.001	532 (39.5%)	240 (17.8%)	130 (54.2%)	48 (20.0%)
**AstraZeneca**	15.4 (38.9)	20.3 (44.6)	9.4 (18.2)	4.6 (19.8)	< 0.001	507 (37.6%)	223 (16.6%)	117 (52.5%)	41 (18.4%)
**Sinopharm**	14.2 (36.5)	18.2 (41.6)	9.6 (24.1)	5.3 (19.0)	< 0.001	504 (37.4%)	228 (16.9%)	148 (64.9%)	36 (15.8%)

^a^We provided the name and the country of origin of the vaccine and did not display any further information (effectiveness, probability of potential side-effects)

^b^Combined first preferences were allowed, hence the sum of the numbers in the column may exceed the sample size

The average WTP results ranged from 14.2 EUR to 30.3 EUR for the different vaccine types. The vaccines were ranked according on the WTP values, and it was observed that consecutive pairings of WTP values showed significant variances in all cases (*p* < 0.001). The highest WTP amount in the vaccinated, registered, but not vaccinated and non-vaccinated subgroups (39.3 (SD = 58.3), 21.0 (SD = 29.2) and 9.7 (SD = 28.8) euros) was indicated for the Pfizer-Biontech vaccine respectively (*p* < 00.1 in all cases). Similarly for the other four vaccines, vaccinated respondents expressed the highest, while non-vaccinated had the lowest WTP values (*p* < 00.1 in all cases).The rate of those who would not pay for the vaccine was highest for the Sinopharm (51.7%) and AstraZeneca (51.2%) vaccines, followed by the Sputnik V vaccine (47.7%). For the Pfizer-Biontech and Moderna vaccines only a third of the respondents expressed that they would not pay for the vaccine (30.2% and 37.0% respectively).

## Discussion

In early 2021, when data were collected, in Hungary both vaccines developed and produced in the EU and one developed and produced in China and Russia were available. We assessed the vaccination in the Hungarian population and analysed factors influencing the vaccine acceptance and individual preferences for the available vaccines using an online cross-sectional survey. The majority of our respondents (69%) has received at least the first dose of vaccine, and the rate of vaccination was higher in the older age (> 65 years) groups (85.6% vs. 63.7%) and among male respondents (73.3% vs.63.7%). Older age (60 +) was associated with higher vaccine acceptance in another study in Hungary [39]. Previous studies reported that women had a lower acceptance towards the vaccine [4, 7] and a study in the Czech Republic also found male respondents were more likely to accept the booster dose than females [40]. Lower vaccine acceptance was associated with lower age and female gender in the UK [6], and younger age in Portugal [41]. Education and income had a positive and significant impact on vaccine acceptance (primary education: 58.2% vs. tertiary education: 79.2%, under median income: 62,0% vs. over median income: 73.8%). Similarly higher education increased vaccine acceptance in Hungary, UK and US [6, 39, 42]; in the UK higher income was associated with higher vaccine acceptance [6]. Presumably, those who were more afraid of COVID-19 infection had decided to vaccinate themselves as FVC-19 scores were higher in the vaccinated and the registered but not vaccinated groups. Lockdown Captivity scores were higher in those respondents who did not receive a vaccine yet. (*p* < 0.001), which may be due to the situation that allowed more relaxed social distancing rules for vaccinated citizens.

﻿﻿﻿In our sample﻿ ﻿the﻿ average willingness to pay results ranged from 14.2 EUR to 30.3 EUR for the different vaccine types. Male respondents, respondents with higher education and income above the median income expressed a higher WTP value for all five vaccines. Vaccinated respondents were willing to pay higher amounts for all vaccines than the registered, but not vaccinated and non-vaccinated. Based on the WTP rankings, most respondent preferred the Pfizer-Biontech vaccine in first place (*n* = 462), and the average difference between the most (Pfizer-Biontech) and less preferred (Sinopham) vaccine was 16.1 EUR. The relatively low mean WTP value (15.4 EUR) for the AstraZeneca vaccine could be partially influenced by the negative news about possible risks and side effects that were dominating the public opinion at the time of our survey [1]. The Eastern vaccines also had a low average WTP score: 16.1 EUR for the Sputnik and 14.2 EUR for the Sinopharm vaccine, which were listed for emergency use presumably due to respondents associating them with a lower quality. Previous research from Malaysia, Indonesia, Chile and Thailand analyzed WTP results and had heterogenous methodology, with different vaccines analyzed. The average WTP values had a broad range, in Malaysia, participants were willing to pay for a dose of COVID-19 vaccine 30.66 USD [43], which is comparable to our results for the Pfizer and Moderna vaccines. In Thailand willingness to pay values ranged from 32 to 46 USD for three different hypothetical vaccines [44], the estimated value in Indonesia was 57.20 USD [23], while participants from Chile expressed high WTP for the COVID-19 vaccine, with a value up to 232 USD [45]. A recent study in four countries (United States, United Kingdom, Spain and Italy) estimated the average value of a hypothetical COVID-19 vaccine which exceeds our results (100–200 USD vs. 48.3 USD, when adjusted for purchasing power parity). Several factors might have contributed to this difference: timing of the study (data was collected much earlier than in our current study), valuation of a hypothetical vaccine [16] and the different healthcare systems and financing in the included countries. These factors may limit comparability along with the methodological differences. 

We were able to rank the different vaccines based on the WTP answers and found that there was a large difference (16.1 EUR) between the first (Pfizer-Biontech) and last ranked (Sinopharm) vaccine’s average WTP value. Another Hungarian study analysed the acceptance of five vaccine types and found that hesitancy is heterogenous by vaccine types: Pfizer and Moderna were more likely to get accepted than Sputnik, followed by AstraZeneca and Sinopharm, which is in line with our WTP rank based preferences [33]. We found that respondents were able to assert their preferences as the majority (71%, *n* = 975) received their first preference of vaccine even though the choice on which vaccine someone could receive was partially limited. 

A methodology related difference may have been present in our WTP results. Each respondent was randomly assigned the first vaccine to value, and significant differences were found in the mean WTP for all five vaccines based on which vaccine the respondent rated first (Kruskal–Wallis H test *p* < 0.001 in all cases). The AstraZeneca, Sputnik V, Sinopharm, and Pfizer-Biontech vaccines had their highest WTP averages in the subgroup of respondents who got them randomly assigned to be rated first. 

Regarding the health policy implications of our findings on the determinants of vaccination can provide information to policymakers to aid in the design of vaccination programs and can help identify factors which are related to non-vaccination, that could be targeted with information campaigns. WTP results for vaccines may also provide input for planning vaccination campaigns with efficient financing potentially involving out-of-pocket payments considering the country’s public health situation. 

This study has limitations that must be addressed. The online data collection method meant that part of the Hungarian population may not have been appropriately represented in our sample, since while the rate of regular internet users has increased in the past two decades (80% in 2019), less, than a third (31%) of the oldest age group (age 65–74 years) used the internet regularly in 2018. Hence, the older age-groups may not have been represented adequately in our sample [46]. Our sample was not representative of the general Hungarian population regarding level of education as respondents with primary education were overrepresented, furthermore due to the data collection method, consent bias might have affected our data (for example: respondents who were more committed to the prevention of COVID-19 may have been more inclined to fill out the questionnaire, while respondents with severe illnesses, or those with poor socioeconomic status may have been less likely to participate).Due to the timing of our questionnaire, other limitations have to be addressed: our survey was conducted in May 2021, when lockdown regulations and restrictions were more relaxed. In Hungary, vaccination required registration, hence respondents with lower online-literacy may have not registered to be vaccinated despite not experiencing vaccine hesitancy. Furthermore, those respondents who were neither vaccinated, nor registered to be vaccinated may have plan to register or receive the vaccine later, which may explain why they expressed higher than zero willingness to pay amount for certain vaccines.

## Conclusion

In conclusion we found that higher level of education, higher income, age, male gender and residence in the capital or county seats were associated with a higher probability of vaccination, suggesting that these groups might be more willing to accept the vaccine. Based on the FCV-19 and GAD-7 scores respondents more frequently reported problems related to mental health effect (fear, anxiety, and disturbed rest), than the physical symptoms of COVID-19 related fear. Lockdown captivity scores indicated that the majority of respondents. We also found that based on the WTP answers the majority of the vaccinated respondents received their first preference of vaccine.

## Data Availability

All data of this study are available from the corresponding author upon reasonable request.

## References

[1] Alammar MA (2021). Ischemic stroke after AstraZeneca (Covid-19) vaccination. Saudi Med J.

[2] Cavaleri M, Enzmann H, Straus S, Cooke E (2021). The European Medicines Agency's EU conditional marketing authorisations for COVID-19 vaccines. The Lancet.

[3] European Centre for Disease Prevention and Control.COVID-19 Vaccine Tracker [https://vaccinetracker.ecdc.europa.eu/public/extensions/COVID-19/vaccine-tracker.html#notes-tab]Accessed 07 July 2023.

[4] Troiano G, Nardi A (2021). Vaccine hesitancy in the era of COVID-19. Public Health.

[5] Razai MS, Osama T, McKechnie DGJ, Majeed A (2021). Covid-19 vaccine hesitancy among ethnic minority groups. BMJ.

[6] Robertson E, Reeve KS, Niedzwiedz CL, Moore J, Blake M, Green M, Katikireddi SV, Benzeval MJ (2021). Predictors of COVID-19 vaccine hesitancy in the UK household longitudinal study. Brain Behav Immun.

[7] Ruiz JB, Bell RA (2021). Predictors of intention to vaccinate against COVID-19: Results of a nationwide survey. Vaccine.

[8] Gilan D, Birkenbach M, Wossidlo M, Sprengholz P, Betsch C, Hahad O, Lieb K (2023). Fear of COVID-19 disease and vaccination as predictors of vaccination status. Sci Rep.

[9] Mertens G, Lodder P, Smeets T, Duijndam S (2022). Fear of COVID-19 predicts vaccination willingness 14 months later. J Anxiety Disord.

[10] Sekizawa Y, Hashimoto S, Denda K, Ochi S, So M (2022). Association between COVID-19 vaccine hesitancy and generalized trust, depression, generalized anxiety, and fear of COVID-19. BMC Public Health.

[11] Neumann-Böhme S, Sabat I, Brinkmann C, Attema AE, Stargardt T, Schreyögg J, Brouwer W (2023). Jumping the Queue: Willingness to Pay for Faster Access to COVID-19 Vaccines in Seven European Countries. Pharmacoeconomics.

[12] Alhassan RK, Nketiah-Amponsah E, Immurana M, Abuosi AA (2022). Financing COVID-19 vaccination in sub-Saharan Africa: lessons from a nation-wide willingness to pay (WTP) survey in Ghana. BMC Public Health.

[13] Arabyat RM, Nusair MB, Al-Azzam SI, Amawi HA, El-Hajji FD (2023). Willingness to pay for COVID-19 vaccines: Applying the health belief model. Research in social & administrative pharmacy : RSAP.

[14] Catma S, Reindl D (2021). Parents' willingness to pay for a COVID-19 vaccine for themselves and their children in the United States. Hum Vaccin Immunother.

[15] Catma S, Varol S (2021). Willingness to Pay for a Hypothetical COVID-19 Vaccine in the United States: A Contingent Valuation Approach. Vaccines (Basel)..

[16] Costa-Font J, Rudisill C, Harrison S, Salmasi L (2023). The social value of a SARS-CoV-2 vaccine: Willingness to pay estimates from four western countries. Health Econ.

[17] Dias-Godói IP (2022). Tadeu Rocha Sarmento T, Afonso Reis E, Peres Gargano L, Godman B, de Assis Acurcio F, Alvares-Teodoro J, Guerra Júnior AA, Mariano Ruas C: Acceptability and willingness to pay for a hypothetical vaccine against SARS CoV-2 by the Brazilian consumer: a cross-sectional study and the implications. Expert Rev Pharmacoecon Outcomes Res.

[18] García LY, Cerda AA (2020). Contingent assessment of the COVID-19 vaccine. Vaccine.

[19] Karam MM, Baki JA, Al-Hajje A, Sraj M, Awada S, Salameh P, Ajrouche R (2022). Willingness to Pay for a Coronavirus Vaccine and Its Associated Determinants in Lebanon. Value in health regional issues.

[20] Le AT, Pham TQ, Nguyen LT, Pham TD, Van Ha N (2022). COVID-19 vaccine acceptance and its determinants among Vietnamese teachers: a web-based cross-sectional survey. AIMS public health.

[21] Lin Y, Hu Z, Zhao Q, Alias H, Danaee M, Wong LP (2020). Understanding COVID-19 vaccine demand and hesitancy: A nationwide online survey in China. PLoS Negl Trop Dis.

[22] Wang J, Lyu Y, Zhang H, Jing R, Lai X, Feng H, Knoll MD, Fang H (2021). Willingness to pay and financing preferences for COVID-19 vaccination in China. Vaccine.

[23] Harapan H, Wagner AL, Yufika A, Winardi W, Anwar S, Gan AK, Setiawan AM, Rajamoorthy Y, Sofyan H, Vo TQ (2020). Willingness-to-pay for a COVID-19 vaccine and its associated determinants in Indonesia. Hum Vaccin Immunother.

[24] Spitzer RL, Kroenke K, Williams JB, Löwe B (2006). A brief measure for assessing generalized anxiety disorder: the GAD-7. Arch Intern Med.

[25] Ahorsu DK, Lin C-Y, Imani V, Saffari M, Griffiths MD, Pakpour AH (2022). The Fear of COVID-19 Scale: Development and Initial Validation. Int J Ment Health Addict..

[26] Martínez-Lorca M, Martínez-Lorca A, Criado-Álvarez JJ, Armesilla MDC, Latorre JM (2020). The fear of COVID-19 scale: Validation in spanish university students. Psychiatry Res.

[27] Satici B, Gocet-Tekin E, Deniz ME, Satici SA (2021). Adaptation of the Fear of COVID-19 Scale: Its Association with Psychological Distress and Life Satisfaction in Turkey. Int J Ment Health Addict..

[28] Reznik A, Gritsenko V, Konstantinov V, Khamenka N, Isralowitz R (2021). COVID-19 Fear in Eastern Europe: Validation of the Fear of COVID-19 Scale. Int J Ment Health Addict..

[29] Tzur Bitan D, Grossman-Giron A, Bloch Y, Mayer Y, Shiffman N, Mendlovic S (2020). Fear of COVID-19 scale: Psychometric characteristics, reliability and validity in the Israeli population. Psychiatry Res.

[30] Soraci P, Ferrari A, Abbiati FA, Del Fante E, De Pace R, Urso A, Griffiths MD (2022). Validation and Psychometric Evaluation of the Italian Version of the Fear of COVID-19 Scale. Int J Ment Health Addict..

[31] Sakib N, Bhuiyan AKMI, Hossain S, Al Mamun F, Hosen I, Abdullah AH, Sarker MA, Mohiuddin MS, Rayhan I (2022). Hossain M *et al*: Psychometric Validation of the Bangla Fear of COVID-19 Scale: Confirmatory Factor Analysis and Rasch Analysis. Int J Ment Health Addict..

[32] Masuyama A, Shinkawa H, Kubo T (2022). Validation and Psychometric Properties of the Japanese Version of the Fear of COVID-19 Scale Among Adolescents. Int J Ment Health Addict..

[33] Kutasi K, Koltai J, Szabó-Morvai Á, Röst G, Karsai M, Biró P, Lengyel B (2022). Understanding hesitancy with revealed preferences across COVID-19 vaccine types. Sci Rep.

[34] Irimiás AR, Mitev AZ (2021). Lockdown captivity: the wish to break out and travel. Curr Issue Tour.

[35] Bowman N, Clark-Gordon C. Fear of Missing Out Scale. In book: Communication Research Measures III. 2019;265–67. 10.4324/9780203730188-29.

[36] Can G, Satici SA (2019). Adaptation of fear of missing out scale (FoMOs): Turkish version validity and reliability study. Psicologia, reflexao e critica : revista semestral do Departamento de Psicologia da UFRGS.

[37] Bobinac A, van Exel NJA, Rutten FFH, Brouwer WBF (2010). Willingness to Pay for a Quality-Adjusted Life-Year: The Individual Perspective. Value in Health.

[38] Central Bank of Hungary. Exchange Rate - May 2021 Monthly Review. http://www.mnbkozeparfolyam.hu/arfolyam-2021-05.html. Accessed 2 Dec 2021.

[39] Dombrádi V, Joó T, Palla G, Pollner P, Belicza É (2021). Comparison of hesitancy between COVID-19 and seasonal influenza vaccinations within the general Hungarian population: a cross-sectional study. BMC Public Health.

[40] Klugar M, Riad A, Mohanan L, Pokorná A (2021). COVID-19 Vaccine Booster Hesitancy (VBH) of Healthcare Workers in Czechia: National Cross-Sectional Study. Vaccines..

[41] Soares P, Rocha JV, Moniz M, Gama A, Laires PA, Pedro AR, Dias S, Leite A, Nunes C (2021). Factors Associated with COVID-19 Vaccine Hesitancy. Vaccines.

[42] Liu R, Li GM (2021). Hesitancy in the time of coronavirus: Temporal, spatial, and sociodemographic variations in COVID-19 vaccine hesitancy. SSM Popul Health.

[43] Wong LP, Alias H, Wong PF, Lee HY, AbuBakar S (2020). The use of the health belief model to assess predictors of intent to receive the COVID-19 vaccine and willingness to pay. Hum Vaccin Immunother.

[44] Prasert V, Thavorncharoensap M, Vatcharavongvan P (2022). Acceptance and willingness to pay under the different COVID-19 vaccines: A contingent valuation method. Research in social & administrative pharmacy : RSAP.

[45] Cerda AA, García LY (2021). Willingness to Pay for a COVID-19 Vaccine. Appl Health Econ Health Policy.

[46] Hungarian Central Statistical Office. STADAT Internethasználók aránya (2005–) [https://www.ksh.hu/docs/hun/xstadat/xstadat_eves/i_int078b.html] Accessed 16 March 2021.

[47] European Centre for Disease Prevention and Control. 2021 Covid-19 Publications and data. https://www.ecdc.europa.eu/en/searchs=&sort_bef_combine=date_DESC&f%5B0%5D=categories%3A1244. Accessed 2 Nov 2021.

